# The transcription factor Blimp-1 is suppressed by SLAMF1 and drives Treg cell-mediated immune evasion in non-small cell lung cancer

**DOI:** 10.1038/s44276-025-00184-9

**Published:** 2025-10-21

**Authors:** Susetta Finotto, Denis I. Trufa, Sonja Trump, Laura Neurath, Katja Hohenberger, Patrick Tausche, Nicole Neurath, Sviatoslav Tsiumpala, Susanne Mittler, Andreas Wild, Elvedina Nendel, Horia Sirbu, Arndt Hartmann

**Affiliations:** 1 Department of Molecular Pneumology, Friedrich Alexander University Erlangen-Nürnberg (FAU), Universitätsklinikum Erlangen, 91054 Erlangen, Germany; 2 Deutsches Zentrum für Immuntherapie (DZI), Erlangen, Germany; 3 Bavarian Cancer Research Center (BZKF), Erlangen, Germany; 4 Comprehensive Cancer Center Erlangen-EMN (CCC ER-EMN), Erlangen, Germany; 5 Department of Thoracic Surgery, University Hospital, Friedrich-Alexander-Universität Erlangen-Nürnberg, Erlangen, Germany; 6 Department of Immune Modulation, Dermatology Clinic, Friedrich Alexander University Erlangen-Nürnberg (FAU), Universitätsklinikum Erlangen, 91054 Erlangen, Germany; 7 Institute of Pathology, University Hospital, Friedrich-Alexander-Universität Erlangen-Nürnberg, Erlangen, Germany

## Abstract

**Background:**

Immune checkpoint inhibitors targeting the interaction between PD1 and PDL1 are effective for immunotherapy in non-small cell lung cancer (NSCLC). However, only subgroups of patients respond to therapy, suggesting the existence of resistance mechanisms.

**Methods:**

In this study, we analyzed post-surgery lung tissues and peripheral blood cells from patients with Non Small Cell Lung Cancer (NSCLC) and control subjects by using western blot, immunohistochemistry, ELISA, multiplex cytokine, and qPCR analysis.

**Results:**

Here, we found progressively increased Blimp1 expression in the tumoral region of NSCLC patients, where Slamf1 significantly decreased, and it inversely correlated with Blimp1. In PBMCs, Blimp1 was expressed in CD8+ T cells but predominantly in immunosuppressive Foxp3+ Treg cells or after targeting of the T cell activator Slamf1. Anti-CD3/CD28 induced IL-6 and IL-10, an immunosuppressive gene induced by Blimp1, in the supernatants of PBMCs from patients with NSCLC. Targeting PD1 in PBMCs reduced Blimp1.

**Conclusions:**

Here, we identify a key role of the transcription factor Blimp1 for immune evasion in NSCLC. Thus, targeting Blimp1 emerges as a novel concept to improve current lung cancer immunotherapies and to suppress immune evasion in lung cancer.

## Introduction

Lung cancer represents the leading cause of cancer deaths worldwide [1, 2]. The illness is classified into two major subtypes: small-cell lung cancer (SCLC) and non-small-cell lung cancer (NSCLC). Despite substantial therapeutic progress in recent years, first-line routine treatments, such as chemotherapy, radiotherapy, and surgery, have demonstrated limited efficacy, and only a subgroup of patients demonstrates remission or progression-free survival over time [3–8]. Resistance to therapy and post-treatment recurrence remain significant causes of morbidity and mortality. Consequently, there is an urgent need for the development of more effective therapeutic options.

Recent studies have highlighted the pivotal role of the immune system in NSCLC. These studies have uncovered the immunogenic nature of lung cancer [9–11]. However, successful elimination of cancer cells by tumor-infiltrating lymphocytes (TILs) is hampered by a series of immunosuppressive mechanisms activated by the tumor and present in the tumor microenvironment (TME) as well as in the peripheral blood [12, 13]. Thus, the ability of tumors to suppress the anti-tumor immune response within the TME is a key phenomenon promoting tumor progression and resistance to therapy. Based on these findings, various types of immunotherapies have been developed to overcome this tumor-mediated suppression of the immune system in order to boost anti-tumor immune responses. Experimental and clinical studies demonstrated that the selective targeting of immune inhibitory T cell surface markers resulted in the activation of anti-tumor T cell responses and suppression of tumor cell growth in NSCLC [14, 15]. Based on controlled clinical trials, the development of antibodies that target the immune checkpoints, programmed cell death 1 (PD1) and programmed death-ligand 1 (PDL1), has revolutionized the clinical treatment of NSCLC. Hereby, immunotherapy can be combined with chemotherapy in order to induce significantly longer event-free survival than chemotherapy in patients with resectable or metastatic NSCLC [9, 16–18].

Despite the significant advancements in immunotherapy for NSCLC, a considerable number of patients experience a progressive course of the disease and succumb to cancer progression. Consequently, it is important to gain a deeper understanding of the molecular mechanisms underlying mucosal effector T cell activation in the lungs of NSCLC patients. Following the activation phase of effector T cells, there is a contraction phase in which the T cells in NSCLC either die or become memory cells [19]. The memory phase is regulated by IL-2, a cytokine that activates intracellular signaling pathways and transcription factors, including PR domain zinc finger protein 1 (PRDM1)/B lymphocyte-induced maturation protein 1, also known as Blimp-1. This transcription factor plays a pivotal role in lymphocyte differentiation [20], regulating both B cell and T cell differentiation. It also affects the homeostasis and function of effector CD4+ and CD8+ T cells, as well as Treg cells [21]. Upon antigen activation, T cells induce Blimp-1 following IL-2 binding to IL-2R. However, at late differentiation phases, Blimp-1 suppresses IL-2 production and increases IL-10 production, thus promoting the terminal differentiation of T cells. Recent studies have demonstrated that IL-2 within the TME facilitates the generation of cytotoxic T helper cells through Blimp-1. Furthermore, Blimp-1 plays a pivotal role in the differentiation of CD8+ T cells into cytotoxic T cells and in the recall response of memory T cells during infections [22].

The regulation of mucosal immune responses and self-tolerance is a key function of Foxp3+ regulatory T cells (Treg). However, the accumulation of these cells within the tumor represents a significant challenge to the development of effective anti-tumor immunity and immunotherapy [23]. A high frequency of Foxp3+ regulatory T cells within the tumor microenvironment (TME) is often associated with a poor prognosis for patients with cancer. Foxp3+ Treg cells are classified according to their phenotypic and functional specialization, with two main subsets identified: central Treg (cTreg) and effector Treg (eTreg) [24]. The eTreg subset displays an activated phenotype and effector program, and expresses Blimp1. Blimp1-expressing Treg cells have recently been proposed as predictors of unfavorable outcomes in certain types of cancer, including melanoma and colorectal cancer [25, 26]. In addition, it has been demonstrated that Blimp1 is essential for the lineage stability and suppressive activity of Foxp3+ Treg cells during ongoing immune or inflammatory responses [27, 28]. Nevertheless, the extent to which they contribute to tumor progression and resistance to therapy in NSCLC remains unclear. In this manuscript, we sought to determine the functional role of Blimp-1 in T cell activation and effector functions in NSCLC.

## Methods

All procedures described below for handling human tissue or blood samples and the preparation and culture of cells were carried out under sterile conditions after an approved ethical application and signed consent of the subjects analyzed in this study.

### Patients with lung cancer

The study is an observational study utilizing all human lung specimens collected. This study was conducted at the Friedrich-Alexander-University Erlangen-Nürnberg, Germany, following approval by the ethics review board of the University of Erlangen (Re-No: 22-64B; DRKS-ID: DRKS00029641). A total of 173 patients diagnosed at the local Pathology department, with primary non-small cell lung cancer (NSCLC), three patients with metastatic (MTS) disease, and 10 Controls (K) underwent surgical procedures after providing written informed consent to participate in this study. The patient study was conducted in accordance with the ethical guidelines in accordance with the Declaration of Helsinki. Patients enrolled in this study did not receive any form of therapy before surgery. The confidentiality of the patients was maintained throughout the study.

The diagnosis of lung cancer was based on pathological confirmation. The histological types of lung cancer were classified according to the World Health Organization (WHO) in 2004. The staging was based on the Cancer TNM Staging Manual, formulated by the International Association for the Study of Lung Cancer (IASLC) and issued in 2010. The clinical data, including the histological classification, TNM stage, age, gender, tumor diameter were provided by the Department of Thoracic Surgery and the Institute of Pathology and are summarized in Tables S1–S3. The clinical data of the control cohort are presented in Table S4. In addition, regarding PBMCs analysis, data of the patients are reported in Table S5 and for the additional control subjects in Tables S6 and S7.

Immediately after surgery, lung tissue samples were taken from three different regions: the tumoral region (TU: solid tumor tissue), peri-tumoral region (PT: 2–3 cm away from the solid tumor), and the tumor-free control region (CTR: >5 cm away from the solid tumor). The post-surgery tissue samples were used for RNA and protein isolation as well as for total cell isolation, followed by cell culture and FACS analysis. Paraffin-embedded lung tissue arrays were generated as previously described [18] and applied for immunohistochemistry (IHC).

### Protein isolation and western blot analysis

Dissected lung tissue was transported on ice immediately after lung surgery to our laboratories and immediately frozen at −80 °C. Later, frozen lung samples were lysed with RIPA Buffer (Thermo Fisher Scientific, Cat# 89900) and inhibitor cocktail (complete Mini EDTA-free, Roche Diagnostics, Cat# 11836170 001), then homogenized with SpeedMill PLUS (Analytik Jena) in innuSPEED lysis Tubes P (Analytik Jena, Cat# 845-CS-1020250) and centrifuged (3000 rpm, 5 min, 4 °C). The supernatant was incubated for 30 min on ice and centrifuged again (2000*g*, 5 min, 4 °C). Final extraction was done by another centrifugation step of the supernatant (45 min, maximum speed, 4 °C) and measured with Bradford Assay (Protein Assay Dye Reagent Concentrate, Bio-Rad, Cat# 5000006), followed by protein denaturation (95 °C, 5 min) in a mix of reducing loading buffer (4xLDS Sample Buffer, Thermo Fisher Scientific, Cat# NP0007) and DTT (1 M, Thermo Fisher Scientific, Cat# P2325). A mini-PROTEAN TGX Stain-Free Gel (Bio-Rad, Cat# 4568086) was used with 20 µg of proteins per well and run at 120 V, 150 mA for 1 h. Proteins were then transferred to a 0,2 µm nitrocellulose membrane (Trans-Blot Turbo Transfer Pack, Bio-Rad, Cat# 1704158) by using a Western Blot Trans-Blot Turbo System (Bio-Rad). Transfer and protein load were assessed and recorded by using a ChemiDoc Imaging System (Bio-Rad). After washing and blocking (3% powdered milk (Carl Roth GmbH, Cat# T145.3) in Tween SDS buffer) the membrane for 1 h at room temperature*,* the membrane was incubated overnight at 4 °C with the primary antibody against Blimp1, cat PA5-20310 (1:500) (Invitrogen) or GAPDH (Cell Signaling Cat 14C10) dissolved in blocking solution. Then the membrane was washed and incubated with the compatible secondary antibody, mouse anti-rabbit-IgG-HRP (sc-2357 Santa Cruz) (1:2000) for both Blimp1 and for GAPDH in blocking buffer, for 1 h at room temperature. After final washing steps, detection was performed using SuperSignal™ West Femto Maximum Sensitivity Substrate (Thermo Scientific, Cat# 34095). Visualization of the western blot results was done by using the ChemiDoc Imaging System (Bio-Rad). Protein bands and total protein levels were analyzed with the ImageLab software (Bio-Rad).

### Immunofluorescence staining for Blimp1 and CD3

Immunohistochemistry was conducted on paraffin-embedded histological sections as described in detail in the Supplementary file and as previously reported [29].

Paraffin sections were melted at 65 °C overnight and then treated 2 × 10 min in Roti-Histol (Roth 6640.4) and followed by 5 min each in descending EtOH (Roth P075.4) from 100%/95%/70% to rehydrate tissue. After this, the slides were washed and pretreated in a demasking solution in 50 ml 1× Citrate-Buffer (TRS Dako) for 5 min at 120 °C. The slides were then left for 30 min at RT and then washed for 5 min in PBS. Unspecific bindings were inhibited by incubation for 1 h in 5% BSA containing Saponin (1:100). After this incubation, the slides were incubated with the primary antibody antiCD3 (Abcam ab699) diluted (1:50) in PBS containing 1% BSA (Serva 11930) and Saponin (1:100) overnight at 4 °C. Negative control was incubated in the same solution without the first antibody. The next day the slides were washed 3× 5 min in PBS followed by 1 h incubation with the secondary antibody AlexaFluor555 (1:500) (F(ab’)2-Goat anti-Mouse IgG (H + L) Cross-Adsorbed Secondary Antibody, Alexa Fluor™ 555; Invitrogen A-21425), washed again in PBS followed by 1 h incubation with anti-Blimp1 (Invitrogen PA5-20310) 1:50 in PBS containing 1% BSA and Saponin (1:100) overnight at 4 °C. After this washed and incubated 1 h with the secondary antibody F(ab’)2-Goat anti-Rabbit IgG (H + L) Cross-Adsorbed Secondary Antibody, Alexa Fluor™ 488 AlexaFluor488 diluted (Catalog # A-11070 Invitrogen) diluted (1:500) in PBS and Saponin (1:100) washed in PBS 3 times for 5 min and mounted with Dapi (Invitrogen D3571) diluted (1:10,000)in Mowiol 4–88 (Roth 0713.1) and covered with a coverslip and stored at 4 °C in the dark until analyzed blindly by SM at the fluorescence microscope Axiovert-Observer D1.Zeiss. The scale bar was inserted after taking the picture by using the Zen Pro software from Zeiss.

### RNA isolation and quantitative real-time PCR (qPCR)

Total RNA was extracted from frozen tissue samples or from cell suspension samples using the Qiazol Lysis® Reagent (QIAGEN, Cat#79306) according to the manufacturer’s instructions. One microgram of the resulting RNA was reverse transcribed into cDNA via the RevertAid™ First Strand cDNA Synthesis Kit (ThermoFisher Scientific, Cat#K1622) according to the manufacturer’s protocol. Each quantitative polymerase chain reaction (qPCR) reaction mixture contained 15 ng of cDNA, 300 nM of transcript-specific forward and reverse primers, and iTaq Universal SYBR Green Supermix (Bio-Rad Laboratories, Cat# 1725124) in a total volume of 20 µl. The qPCR primers were procured from Eurofins-MWG-Operon (Ebersberg, Germany). The primer sequences for the human glyceraldehyde-3-phosphate dehydrogenase (GAPDH) gene are provided in the QuantiTect® Primer Assay.

### Quantitative real-time PCR (qPCR)

The primer sequences for human qPCR analysis are presented in the Supplementary Table S9. The reactions were conducted for 50 cycles, with an initial activation for 2 min at 98 °C, denaturation for 5 min at 95 °C, and hybridization and elongation for 10 min at 60 °C. Quantitative polymerase chain reaction (qPCR) reactions were performed using the CFX-96 Real-Time PCR Detection System (BIO-RAD, Munich, Germany) and analyzed via the CFX Manager Software. The relative expression level of specific transcripts was calculated using the relative quantification 2-ΔΔCT method with respect to the internal standard glycerinaldehyd-3-phosphat-dehydrogenase (GAPDH) and Hypoxanthin-Guanin-Phosphoribosyl-Transferase (HPRT). Data with ΔCq values higher than 35 were excluded [29, 30].

### PBMCs isolation

After providing written informed consent to participate in this study, blood samples were collected from donors while in 7.5 ml K3 EDTA vacutainer (Sarstedt, Germany) for all NSCLC patients or in 9 ml K3 EDTA vacutainers for all healthy subjects. To obtain peripheral blood mononuclear cells (PBMC) from the blood of patients or control subjects, donated blood was mixed with an equal volume of PBS and applied to a leucosep tube (Greiner Bio-one, Cat# 227290 Kremsmünster, Austria) according to the manufacturer’s protocol with separation by Biocoll (Bio & Sell, Cat# 6715, Feucht, Germany) and centrifuged (30 s, 1000×*g*, RT) without brakes. Thereafter, the PBMC layer was carefully removed and washed with RPMI-1640 medium (Anprotec, Bruckberg, Germany) by centrifugation at 250*g* and room temperature (RT) for 10 min. Cell pellet was washed twice with RPMI 1640 (centrifugation at 200*g*, 15 min, RT). Erythrocytes removed by resuspending PBMC in 5 ml of ACK-lysis buffer followed by immediate centrifugation at 1500 rpm, 5 min, 4 °C. Pelleted PBMC were resuspended in supplemented medium (RPMI 1640, 10% FCS, 1% L-Glutamine, 1% Penicillin/Streptomycin) for cell counting (Neubauer chamber) and subsequent applications.

### PBMCs cell culture with anti-CD3/anti-CD28 antibodies

Following cell counting with Trypan Blue in a Neubauer counting chamber, the PBMCs were cultured for 4–5 days with 5 × 10^5^ cells per condition. A total of 3 healthy controls (HC) and 6 non-small cell lung cancer (NSCLC) patients (1 with metastasis) were isolated and cultured at a density of 5 × 10^5^ cells/well. The cells were cultured in the presence of plate-bound anti-CD3 (10 µg/ml) and soluble anti-CD28 antibodies (10 µg/ml), with and without transforming growth factor beta (TGFβ1; 20 ng/ml).

### PBMCs culture with anti-CD150 and anti-PD1 antibodies

The study on the PBMCS cultures with and without anti-PD1 and anti-CD150 antibodies was performed in four control subjects recruited under the study AZCRA approved by the local ethics committee of the Universitätsklinikum at the Friedrich–Alexander-Universität Erlangen–Nürnberg (FAU), Germany (Re-No. 315_20B). The study is registered in the German Clinical Trial Register (Deutsches Register Klinischer Studien: registration No. DRKS00023843). Informed consent was obtained from all participants included in the study. From the obtained whole blood samples, we isolated human PBMCs and cultured them with and without antiCD3/CD28 antibody stimulation and with and without anti CD150 (Abcam Ms mo AB SLA; CD150 (IPO-3) (5 micrograms/ml); cat AB183269) or IgG1 (BioXCell (In vivo Mab; MOPC21, cat BE0083) (5 µg/ml) or anti PD1 antibody BioXCell (In Vivo Mab, J116 BE0188) (5 µg/ml). After 24 h, RNA was collected and extracted, and later on, we analyzed Blimp1, CD25, and HPRT via qPCR. The cell supernatants were analyzed after 24 and 48 h of cell culture with Multiplex cytokine assays by FACS. PBMCs from 3 additional control people from the same AZCRA study were analyzed for the investigation on IL-6R chain expression on T cells.

### Flow cytometric analysis of human cells

The antibodies utilized in this study for human FACS analysis are listed in Table S8. The data sets were analyzed using FlowJo v10.2 software (FlowJo, LLC, OR, USA) and Kaluza.

### Multiplex cytokine assays

To determine the protein concentration of IL-2, IL-6, and IL-10 in the medium supernatant of human PBMC cell cultures, the LEGENDplex™ HU Th Cytokine Panel (12-plex), Cat# 741028, was used in accordance with the enclosed protocols. For this purpose, a mixture of 5 µl assay buffer, 5 µl detection antibody, and 5 µl capture beads was pipetted into each well of the enclosed V-bottom plates, which were supplied with 5 µl of the respective sample. After 2 h of incubation at room temperature in the dark, 5 µl of the streptavidin-PE solution was added to each well and incubated for 30 min. In total, 150 µl wash buffer was added to each well, and the well plates were centrifuged at 2500 rpm for 5 min at RT. The supernatant was then carefully tilted, and the samples were taken up in 100 µl wash buffer for immediate analysis at the flow cytometer. A standard series for concentration determination was prepared according to the kit protocol.

### ELISA

The DuoSet™ ELISA Development System R&D Human SLAM/CD150 Catalog Number: DY164 was used for the detection of soluble CD120 (Slamf1), and TGFβ1 Cat number DY240-05 for the detection of TGFβ1, according to the manufacturer’s recommendation.

### Statistical analysis

The significance of the differences was evaluated using GraphPad, with the following levels of significance: *P < 0.05; **P < 0.01; ***P < 0.001; ****P < 0.0001. The data were imported into a column statistics program and subsequently analyzed for normal distribution using the Shapiro–Wilk test and Kolmogorov–Smirnov test. The statistical test was selected according to whether the data exhibited a normal distribution or a non-normal distribution. In the case of normal distribution, a one-way ANOVA with multiple comparisons was employed. In the event of a non-normal distribution or a sample size of less than five, the Kruskal-Wallis test was employed. In the case of two independent columns and a normal distribution, the unpaired *t*-test was employed. In the case of a non-normal distribution, the Mann–Whitney test was selected. Correlations were examined by importing data into tables and by using linear regression curves. A two-tailed Pearson correlation analysis was conducted to obtain the correlation coefficient (*r*) and the associated *p*-value. *P*-values were adjusted, and post hoc analysis was performed using the Bonferroni–Holm correction. The survival data are presented in Kaplan–Meier survival curves. The log-rank test was employed to compute *P*-values. The data are presented as mean values ± standard error of the mean (SEM). The graphs were created using GraphPad Prism 8 (Windows).

In this study, cell percentages were accurately determined using FlowJo (version 10.9) or Kaluza software, with further statistical analysis performed in GraphPad Prism 9, presenting results as mean ± SEM. For two-group comparisons, the unpaired Student’s two-tailed *t*-test or the Mann–Whitney test for normally and non-normally distributed data, respectively, were applied. One-way ANOVA was used for analyzing multiple groups based on a single independent variable, while two-way ANOVA was employed for data involving two independent variables, including their interaction effects. Additionally, correlations were discerned through simple linear regression. A *P*-value below 0.05 signifies statistical significance.

## Results

### Blimp1 protein is induced in the immunosuppressive tumor microenvironment of the lung of patients with NSCLC

Immunotherapy has the potential to improve the prognosis for lung cancer by activating cytotoxic CD4+ and CD8+ T cells in the immunosuppressive tumor microenvironment and concomitantly suppressing Treg cell activation [31–35]. Previously, we demonstrated that the recognition and elimination of lung tumor cells by T lymphocytes requires the presence of T-box expressed in T cells (Tbet), a transcriptional regulator that induces Th1 differentiation, promotes cytotoxic CD8+ T cells, NK and NKT cells, and inhibits Treg cells [18]. As both T-bet and the transcriptional repressor B lymphocyte-induced maturation protein-1 (Blimp1) have been shown to orchestrate CD8+ T cell effector functions [36], and as Blimp-1 also affects the activation of CD4+ as well as Treg cells [37–39], we postulated that this transcription factor might play an important role in driving immune responses to lung cancer in NSCLC patients. Consequently, we initiated an investigation into the potential role of Blimp-1 in NSCLC.

**Fig. 1:**
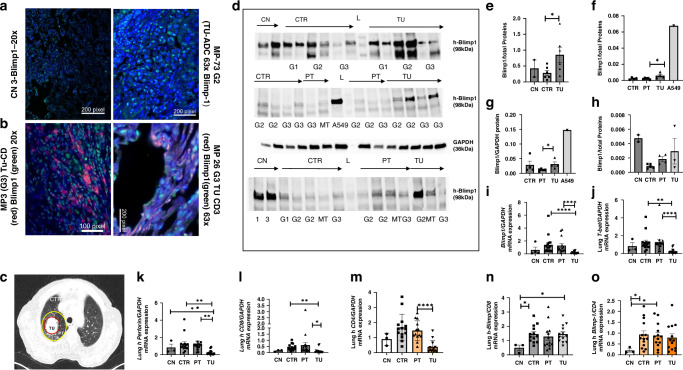
Blimp1 is induced in the tumoral region of the lung from patients with lung adenocarcinoma (LUAD). **a** Representative Blimp1 immunofluorescence (IF) staining on histologic sections in the lung from a control subject (CN) and from a patient with lung adenocarcinoma (ADC). TU tumoral region. Higher expression of Blimp1 (in green) was noted in the TU region of patients with lung adenocarcinoma (ADC) as compared to a control (CN) subject. Blimp1+ cells are green in the image from the lung of a CN subject (left panel) and from the tumoral region of the lung of a NSCLC patient with tumor grade G2 having a tumor diameter of 4.8 cm, stage IIA at surgery (right panel). The nuclei are counterstained in blue with DAPI staining on the mounting medium. **b** Double IF for Blimp1 and CD3 showing co-localization in the tumoral region of the lung of patients with NSCLC. Blimp-1 positive cells (green) were co-localized in the lung tumor microenvironment with CD3+ positive T cells (red stained). Co-localization of CD3+ (red) with Blimp1 (green nuclei) is shown. Nuclei were counterstained with DAPI. Pictures’ magnifications are indicated below the microscopic picture. (MP Molecular Pneumology) MP-3 = subject 3 Grading G3 and stadium IIA having a tumor of 5 cm in diameter at surgery (Table S1). In the right picture, the same staining in a patient with NSCLC, Grading G3 and Stage IVA, with a tumor diameter = 1.3cm at surgery. **c** A computer tomography of a human right thorax with lung non-small cell lung cancer. Schematic drawing of the regions where tissue samples were collected and dissected for further analysis: tumoral area (TU) (red circle), peritumoral area (PT) (2 cm around the tumor) (yellow circle), and control region (CTR) (>5 cm away from the tumor). **d** Immediately after lung surgery, lung samples from control subject CN = lung and from NSCLC patients (histologically classified as ADC) were dissected from the tumor area (TU), the peri-tumoral area (PT) surrounding the tumor, and from the control area (CTR). Western blot analysis of total proteins extracted from NSCLC lung tissue samples including CN (patients without tumor), CTR (control area), PT area and TU (tumoral area) is shown. Luminescence-mediated analysis using a polyclonal antibody against Blimp1 was performed, and the Blimp1 protein was detected at around 98 kDa, and GAPDH at 36 kDa. **e** Western blot analyses for Blimp-1 in lung tissues were normalized for total sample proteins and are shown as mean values ± SEM. Expression Blimp1 protein analysis of the 3 western blots from the top left to right (**e**–**h**), respectively. Abbreviations: Carcinoma histologic grading G1 (Low grade): well-differentiated—cells look similar to normal and tend to grow slowly. Specifically for Adenocarcinoma G1: ≤20% high-grade histologic patterns (lepidic predominant); G2 (Intermediate grade): Moderately differentiated—cells have noticeable abnormalities and a moderate growth rate. Adenocarcinoma : G2: 5–50% intermediate patterns (acinar/papillary); G3 (High grade): Poorly differentiated—cells look very abnormal and often grow quickly; adenocarcinoma:G3: >20% high-grade patterns (solid/micropapillary predominant). MT metastasis. **i** Analysis of Blimp1/GAPDH mRNA extracted from the lung of subjects with NSCLC and from the CTR, PT, and TU regions of patients with NSCLC. Detailed single results are reported in Supplementary data. Like Blimp1(**i**), also T-bet (**j**) and Perforin/GAPDH (**k**), CD8/GAPDH (**l**), and CD4/GAPDH (**m**) mRNA expression were found to be downregulated in the tumoral region of the lung as compared to the peri-tumoral region of the lung in NSCLC patients. **n**, **o** However, the ratio Blimp-1/CD8 and Blimp-1/CD4 mRNA was not downregulated, and the ratio Blimp-1/CD8 was even found to be significantly upregulated in the tumoural region (CN: *n* = 3; groups of ADC patients: *n* = 13–16). Data are shown as mean values ± SEM using Student's two-tailed *t* test. *p < 0.05; **p < 0.01; ***p < 0.001.

In initial studies, we analyzed the role of Blimp-1 in NSCLC patients by taking advantage of resected lung specimens. The expression of the Blimp1 protein was analyzed in the tumorous and control regions of the lung in post-surgical tissue samples from a cohort of patients with NSCLC and control patients. Lung sections from NSCLC patients were fixed and immune-stained with either anti-CD3 or anti-Blimp1 antibodies (Fig. 1a, b). Blimp-1-positive cells were observed to localize in the vicinity of the tumor in NSCLC patients. In this tumor region also Blimp+ CD3+ cells where CD3-positive T cells were detected (Fig. 1b). Furthermore, analyses of large patient cohorts using the Cancer Genome Atlas Program (TCGA) and Kaplan–Meier plot (KMplot) analysis demonstrated that NSCLC patients with elevated mRNA expression levels of PRDM1, the gene encoding Blimp1, exhibited significantly reduced overall survival (OS) in comparison to NSCLC patients with low PRDM1 mRNA expression levels (Supplementary Fig. S1), suggesting that Blimp-1 expression may influence tumor progression.

In subsequent studies, we employed western blot analysis to quantify Blimp1 protein expression in three distinct regions of the lung of NSCLC patients: the control, the peri-tumoral, and the tumoral regions. In these studies, we performed Western blot analysis using proteins isolated from the lung of a cohort of patients who underwent surgery for NSCLC and subsequent histological classification as lung adenocarcinoma (LUAD). The results demonstrated that patients with LUAD exhibited an increase in Blimp1 protein expression in the tumor region (TU) of the lung, as compared to the control region (CTR), which was tumor-free. Blimp1 was predominantly expressed in the LUAD lung region (Fig. 1d–h). Nevertheless, Blimp1 protein expression was also observed in control lung tissue from patients without tumors (CN) (Fig. 1d–h, i), which is consistent with a potential homeostatic function of Blimp1 in T cells.

We conducted further analysis of Blimp1 and Tbet mRNA expression in these patients and found that both Blimp-1 and T-bet mRNA levels were predominantly expressed in the control and peri-tumoral regions of the lung and downregulated in the tumoral region (Fig. 1i, j, respectively). Thus we reasoned that the increase of Blimp1 at protein and not mRNA level in the tumor region could be that RNA is generally more unstable than proteins in tumor tissue—both biologically and technically. Moreover, Blimp1 is a folded and stable protein under physiological conditions, as is generally necessary for it to function as a transcriptional repressor. Infact it contains structured (folded) domains, notably: a SET domain-like PR domain (for protein-protein interactions and repression activity), five C2H2 zinc finger motifs that bind specific DNA sequences. These domains are well-folded, enabling specific binding to DNA and interaction with co-repressors. Its zinc finger domains in particular require proper folding to coordinate zinc ions and maintain structural integrity. In summary we considered of significance the upregulation of Blimp1 protein in the tumor region of the lung of patients with NSCLC. The definition of T cell exhaustion encompasses the diminished functionality of effector T cells following prolonged exposure to a high antigen load [40]. Given that perforin is the mediator enabling T cells to destroy tumor cells, quantitative polymerase chain reaction (qPCR) for perforin was conducted on the same lung samples, revealing a reduction in expression in the tumor region of the lung of patients with non-small cell lung cancer (NSCLC) (Fig. 1k).

To further investigate the relationship between Blimp1 and T cells in lung cancer, we analyzed perforin, CD4, and CD8 mRNA expression. Similarly, we observed an upregulation of CD8 mRNA expression in the control region as compared to the tumoral region of patients with LUAD (Fig. 1l). Furthermore, CD4 mRNA was found to be downregulated in the tumor region as compared to the peri-tumoral region of the lung in patients with LUAD (Fig. 1m). The ratio of Blimp-1 to CD8 and CD4 (Fig. 1n, o) was significantly elevated in the lung of patients with LUAD as compared to the ratio observed in the lung of control subjects. Moreover, although we did not analyze this in the lung tissue of lung adenocarcinoma subjects (LUAD), it has been demonstrated that tumor-infiltrating effector Tregs (eTregs) within the tumor immune microenvironment express high levels of Blimp1, which is encoded by *Prdm1*. These eTregs are also Foxp3^+^—meaning Blimp1 identifies a Foxp3-expressing Treg subpopulation in tumors [26, 41].

### Slamf-1 is decreased in the tumoral region of the lung of patients with NSCLC, where it is directly correlated with Blimp1

**Fig. 2:**
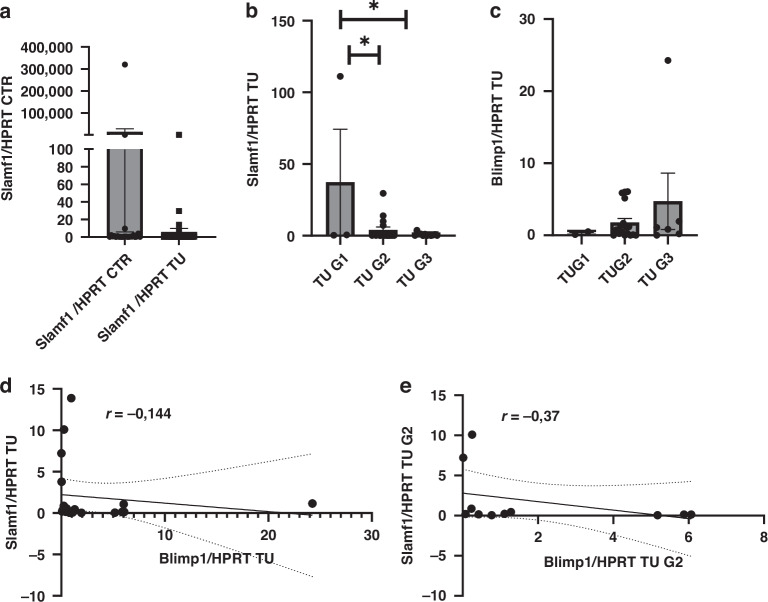
In the tumor region of the lung, Slamf1 mRNA is downregulated in the tumoral region as the tumor progresses. **a**–**c** In additional subjects recently newly recruited, enclosed in Table S1, we measured lung Slamf1 and Blimp1 mRNA expression in the tumoral and control region. Here we found that Slamf1/HPRT mRNA is decreased significantly in the tumoral region as the tumor progresses from G1 to G2 and G3 (G = Histologic grade of the tumor) (**a**, **b**). By contrast, Blimp1/HPRT increased by trend in these patients in the tumoral region from G1 to G3 (**c**). Moreover, Blimp1/HPRT inversely correlated with Slamf1/HPRT in the TU region of the lung (*n* = 20: G1 + G2 + G3), and this kind of correlation was also seen in these patients with tumor grade G2 (n = 11) (**d**, **e**). Data are shown as mean values ± SEM using Student two-tailed *t*-test. *p < 0.05; **p < 0.01; ***p < 0.001.

In order to identify factors controlling Blimp-1 expression, we focused on the glycoprotein SLAMF1 (CD150), which is expressed on lymphocytes and acts as an important co-stimulatory molecule [42–44]. We next analyzed Blimp-1 and Slamf1 mRNA expression in the lung of NSCLC patients in the control and tumoral regions (Fig. 2a–c). A trend towards decreased Slamf1/HPRT mRNA expression was observed in the tumoral regions of the lung of patients with NSCLC, when compared to the control region (Fig. 2a). We next asked if Slamf1 and Blimp1 mRNA expression in the tumoral region would be influenced by the tumor grade (Fig. 2b, c). Here we found a significant downregulation of Slamf1 as the tumor progresses from G1 to G2 and G2 to G3 (Fig. 2b). By contrast, Blimp1 demonstrated an opposite trend, increasing in the tumoral region of more progressed tumor kind (Fig. 2c). We next proceeded to correlate the expression of Slamf1 and Blimp1 mRNA (Fig. 2d, e). Although no significant correlation was found, an inverse correlation trend was evident (Fig. 2d, e). This led us to hypothesize that Slamf1 may be counteracting Blimp-1 expression. However, the exact relationship of these two factors remains to be explored.

### Blimp1 is induced in CD3+ CD8+ T cells as well as in CD4+ CD25+ Foxp3+ T regulatory cells obtained from PBMCs of patients with NSCLC after anti-CD3/CD28 stimulation and TGF-beta1

**Fig. 3:**
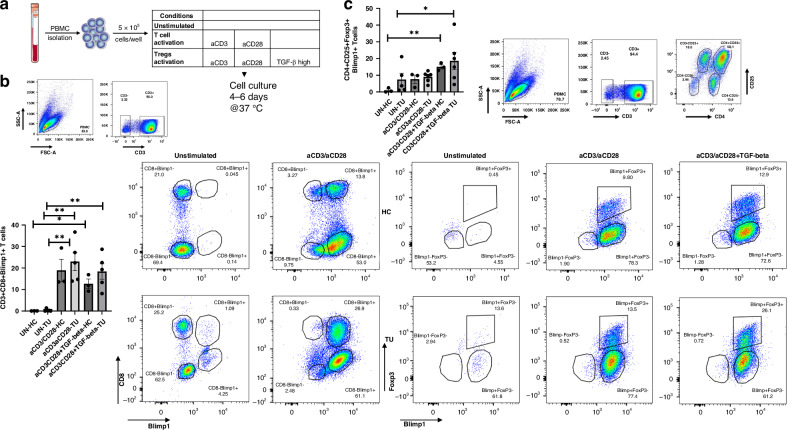
Anti-CD3/CD28-treated PBMCs from patients with NSCLC showed increased numbers of Blimp1+ T regulatory cells. **a** Peripheral blood mononuclear cells (PBMCs) from healthy control (HC), control Subject (CN) and from NSCLC patients were isolated for cell culture studies. Cells were left unstimulated (UN) or stimulated with anti-CD3/CD28 antibodies in the presence or absence of TGF-beta. **b** The percentage of CD3+ Blimp-1+ cells was determined by FACS analysis. Anti-CD3/CD28 antibodies induced Blimp1+ CD8+ T cells in G2 or G3 grade (representing the tumor grade shown in Supplementary Table S5) tumor patients. **c** The percentage of CD3+ CD4+ FoxP3+ Blimp-1+ cells was determined by FACS analysis. Anti-CD3/CD28 antibody treatment with TGF-beta1 induced Blimp1+ CD4+ CD25+ Foxp3+ Tregs in PBMCs obtained from healthy controls as well as from NSCLC patients. Data are shown as mean values ± SEM using Student’s two-tailed *t* test. *p < 0.05; **p < 0.01; ***p < 0.001.

We then proceeded to analyze the expression of Blimp1 in peripheral blood mononuclear cells (PBMCs) derived from patients with non-small cell lung cancer (NSCLC) with histologic grade G2 or G3. Consequently, we conducted a flow cytometry analysis of peripheral blood mononuclear cells (PBMCs) from patients with non-small cell lung cancer (NSCLC) and healthy controls (HC) after culturing the cells without and with anti-CD3/CD28 antibody stimulation with and without TGF-beta1 (Fig. 3a). In the NSCLC patient group, we observed an induction of Blimp1+ CD3+ CD8+ T cells following anti-CD3/CD28 stimulation (Fig. 3b). Moreover, adding TGF-beta to the cell culture did not influence the number of  CD3+CD8+ T cells in these subjects. We then proceeded to examine Foxp3+ CD3+ CD4+ CD25+ regulatory T cells (Tregs) and observed that anti-CD3/CD28 antibody stimulation along with TGFβ, an inducer of Treg cell development, induced Blimp-1+ Foxp3+ Treg cells, in both groups (Fig. 3c). We therefore sought to examine the function of Blimp1 in Treg cells.

### IL-6, an inhibitor of Foxp3, and the immunosuppressive cytokine IL-10 are induced in PBMCs of patients with NSCLC after anti-CD3/CD28 antibody stimulation

**Fig. 4:**
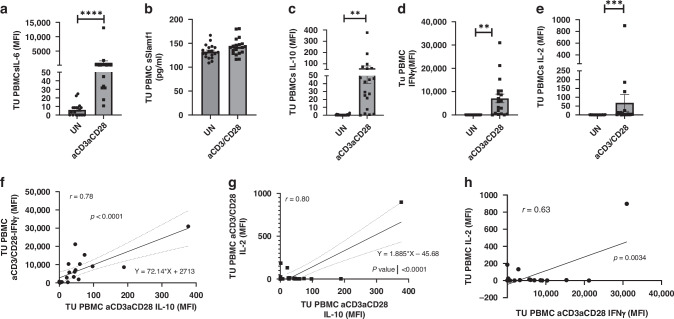
IL-6 and IL-10 are induced after anti-CD3/CD28 stimulation in PBMCs from patients with NSCLC. **a** PBMCs supernatants from 19 NSCLC patients were collected. Cells were either untreated (UN) or treated with anti-CD3/CD28 antibodies. The **a**–**e** levels of IL-6, IL-10, IFN-gamma, and IL-2 in supernatants from PBMCs of patients with NSCLC were determined by multiplex analysis. The level of sSlamf1 was measured by ELISA. **f**–**h** Correlation analysis between IFN-γ and IL-10, IL-2 and IFN-γ, and between IL-2 and IL-10 levels in supernatants. It was found that, after activation of PBMCs from NSCLC patients, the immunosuppressive cytokine IL-10 correlated with levels of the effector cytokines IL-2 and IFN-gamma. These two latter cytokines correlated as well. Data are shown as mean values ± SEM using Student’s two-tailed *t* test. *p < 0.05; **p < 0.01; ***p < 0.001.

We next reasoned that IL-6 has been shown by us and others to inhibit Foxp3. [45] We thus analyzed IL-6 (Fig. 4a) and soluble Slamf1 (Fig. 4b) in the supernatants of PBMCs of patients with NSCLC without and with anti-CD3/CD28 antibody stimulation and found that IL-6 but not soluble Slamf1 is induced under the same conditions after anti-CD3/CD28 stimulation. However, in these supernatants, the immunosuppressive cytokine IL-10 (Fig. 4c) was found to be upregulated in the supernatants of PBMCs of patients with NSCLC by anti-CD3-CD28 antibody stimulation.

It has been previously reported that the ratio of IFNγ and IL-10-producing CD4+ T cells can predict the response to anti-PD1 immunotherapy in melanoma [46]. We thus measured IFN-gamma in the supernatants of PBMCs derived from patients with lung tumors, with and without anti-CD3/CD28 antibody stimulation. Here we found that after anti-CD3/CD28 stimulation, both cytokines were elevated (Fig. 4c, d). Moreover, since IL-2 is a growth factor for both T effector and T regulatory cells we measured in the same supernatants also IL-2 which was also found elevated after anti CD3/CD28 stimulation (Fig. 4e). Moreover, levels of both IFN-gamma and IL-2 directly correlated with IL-10 levels after activation (Fig. 4f, g) and a weaker correlation was found between IL-2 and IFN-gamma (Fig. 4h). The direct correlation between IFNγ and IL-10 is in agreement with a potentially poor prognosis after anti-PD1 immunotherapy [46], as the beneficial effect of IFNγ might be counteracted by IL-10, whose expression is induced by the transcription factor Blimp-1 [47].

### IL-6R is induced in PBMC by anti-CD3CD28 antibodies in Blimp1+ T regs

**Fig. 5:**
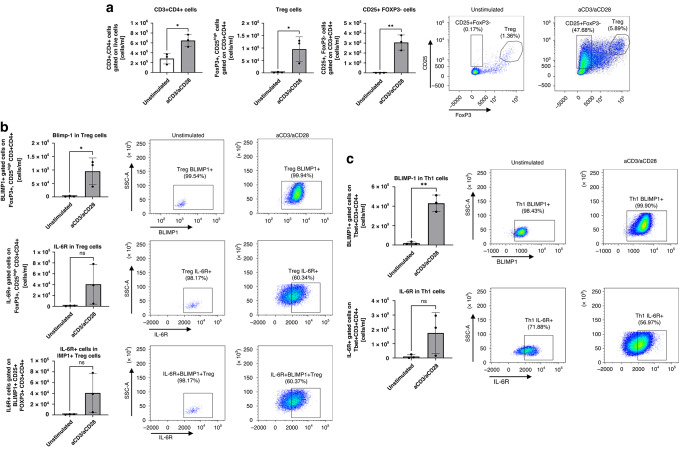
Anti-CD3/CD28-treated PBMCs from control subjects showed increased numbers of Blimp1+ T regulatory cells bearing IL-6R. **a**–**c** In an additional experiment, we looked at IL-6R expression in T regs and Th1 cells expressing Blimp-1 in 3 control subjects. Here we found that 50% of the T reg and Th1 cells expressed IL-6R and Blimp-1. Th1 cells were identified as CD3⁺ CD4⁺ T-bet⁺ cells (gating not shown). Tregs were defined as CD3⁺ CD4⁺CD25⁺ FoxP3 high cells. Data are shown as mean values ± SEM using Student’s two-tailed *t* test. *p < 0.05; **p < 0.01; ***p < 0.001.

Because IL-6 is an inhibitor of Foxp3 [45, 48] and Blimp1 induces Foxp3 stabilization [49], we wanted to analyze the distribution of IL-6R on Blimp + T regs and compare their number with the number of T-bet+ IL-6R+ Blimp1+ T cells. To this aim, we isolated PBMCs from 3 additional control subjects and cultured them with and without anti-CD3/CD28 for 48 h and looked for IL-6R expression in T-regs and Th1 Blimp1+ (Fig. 5a). Th1 cells were identified through a sequential gating strategy: single cells were first selected based on forward and side scatter parameters (FSC-A vs. FSC-H), followed by gating on lymphocytes using FSC vs. SSC. Viable cells were then selected by excluding dead cells using a viability dye. CD3⁺ CD4⁺ T cells were gated, and Th1 cells were defined as T-bet⁺ cells within the CD3⁺CD4⁺ population. Here, we found that circa 100% of T reg Blimp1+ and only 50% of the Th1 Blimp+ cells expressed IL-6R after anti-CD3/CD28 antibody challenge (Fig. 5b, c). This suggests a strong connection between IL-6R expression on the cell membrane and Blimp1 expression on T regulatory cells that needs further investigation.

### Slamf1 neutralizing antibodies induced Blimp1 expression and increased IL-10 production in PBMCs

**Fig. 6:**
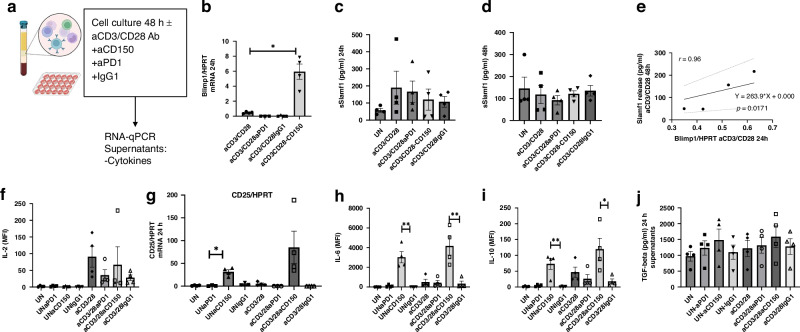
Blocking PD1 function reduced Blimp-1 mRNA expression, while blocking Slamf1 function resulted in an induction of Blimp-1 mRNA levels and the immunosuppressive cytokine IL-10 in PBMC. **a** Experimental design in cell culture using PBMCs (48 h). Slamf1 function was blocked with anti-CD150 antibodies, while PD1 function was targeted with anti-PD1 antibodies (*n* = 4 patients). **b** Blimp1 mRNA expression was analyzed by qPCR and normalized by HPRT analysis. While anti-PD1 antibody treatment reduced Blimp-1 levels, anti-CD150 antibody treatment markedly induced Blimp-1 expression. **c**, **d** Release of soluble Slamf1(measured by ELISA) in different experimental conditions after 24 and 48 h culture. **e** Significant positive correlation between soluble Slamf1 protein and Blimp-1 mRNA expression. **f** Production of IL-2 after different stimulations was measured by multiplex analysis. **g** Levels of CD25/HPRT mRNA were measured in different experimental conditions. **h**, **i** IL-6 and IL-10 production in different experimental conditions were measured by multiplex analysis. **j** Activated TGF-beta was measured after activation in the supernatants by ELISA. Data are shown as mean values ± SEM using Student’ two-tailed *t* test. *p < 0.05; **p < 0.01; ***p < 0.001.

To ascertain the potential relationship between Slamf1 and Blimp-1, we neutralized the function of Slamf1 in PBMCs from control subjects using anti-Slamf1 antibodies. In addition, we inhibited PD1, an inhibitory receptor commonly employed in current immunotherapy for NSCLC and a factor induced by Blimp1 (Fig. 6a) [50, 51]. As a negative control, we utilized the isotype control IgG1. The results demonstrated that treatment with anti-PD1 antibodies decreased anti-CD3/CD28-induced Blimp-1 expression, while anti-Slamf11/CD150 antibody treatment significantly augmented anti-CD3/CD28-induced Blimp-1 levels (Fig. 6b), suggesting that Slamf1 suppresses Blimp-1 expression. In addition, soluble Slamf1 was found to be induced by anti-CD3/CD28 and to directly correlate with Blimp1 levels (Fig. 6c–e) in PBMCs stimulated by anti-CD3-CD28, suggesting a functional relationship between Blimp1 and Slamf1. Although the role of soluble Slamf1 is not entirely clear, an inhibitory function in contrast to the membrane-bound Slamf1 form has been suggested. Here, we found that anti-CD3-CD28 antibody treatment induced soluble Slamf1 levels in PBMCs, which could not be counteracted by anti-PD1 nor anti-CD150 antibody treatment (Fig. 6c). Similarly, interleukin-2 (IL-2), a T effector as well as a T regulatory T cell (Treg) growth factor [52], was found to be induced by anti-CD3/CD28 stimulation in PBMCs (Fig. 6f). Furthermore, we observed that only anti-Slamf1 antibodies induced CD25 mRNA (Fig. 6g), IL-6 (Fig. 5h) and IL-10 (Fig. 5i) and TGFbeta (by trend) (Fig. 6j) production, both with and without anti-CD3/CD28 antibody treatment. In conclusion, our findings demonstrate that inhibiting Slamf1 results in increased Blimp1 expression and augmented IL-2Ralpha (CD25), IL-6, IL-10, and TGFbeta production (Fig. 6f–j), which is consistent with the hypothesis that Slamf1 controls Blimp1 and Treg activation. This led us to propose the hypothesis that the inhibition of Blimp1 via anti-PD1 treatment or Slamf1 overexpression may serve to inhibit the Blimp1-mediated T regulatory cell stabilization observed in cancer [53].

## Discussion

The cellular components of the tumor microenvironment are of critical importance with regard to the growth and expansion of tumors. In this study, we have found induction of Blimp1 protein in the tumor region of the lungs of patients with NSCLC. Moreover, we found that Blimp1 mRNA expression was induced in the tumoral region of the lung with an increase in the tumor histopathologic grade. Finally, we provided evidence of co-localization of Blimp1 and CD3+ T cells in the tumor region of patients with advanced-grade NSCLC. These data suggest that the tumor microenvironment induces Blimp1 in T cells. Blimp1 has been identified to belong to a cluster of transcription factors like T-bet associated with Tissue-resident T (TRM) cells carrying CD103, which keeps the TRM by the tumor, via the ligand of E-Cadherin on the epithelial cells. Moreover, these TRM express PD1 and are located near the tumor cells, which are of Epithelial origin [54]. Thus, it has been suggested that, during current anti-PD1 immunotherapy in lung cancer, these TRM are activated to secrete cytotoxic mediators against lung cancer cells. Furthermore, in this paper, we provided evidence that, not only in the tissue but also in the peripheral blood, in the PBMCs of patients with NSCLC, not only CD8+ T cells but also CD4+ CD25+ Foxp3+ T regulatory cells express Blimp1, and these T reg cells are induced by the immunosuppressive cytokine TGFbeta1.

Treg cells have been demonstrated to exhibit remarkable stability, retaining their identity and function in a range of conditions, both during steady state and in inflammatory and tumor-associated contexts [55]. A variety of signals, including costimulatory signals, were identified to contribute to the stability of Treg cells [56]. However, Treg cells lose their identity upon the loss of Foxp3 expression [57]. Blimp1 is a major regulator of IL-10 in Treg cells [58] and is a factor required to maintain Foxp3 expression. Notably, IL-10 derived from conventional T cells is required to induce full suppressive effector functions in Treg cells. A previous work provided a mechanistic underpinning for cytokine-guided stability or plasticity of Treg cells by linking cytokine signaling pathways to the Blimp1-mediated transcriptional modulation of the enzymatic machinery that controls DNA methylation in *cis-*regulatory elements of the *Foxp3* locus. Tet family enzymes and Dnmts are opposing players in demethylating or methylating CpG islands within DNA elements in immune cells and non-immune cells [59]. Following up on this concept, we thought that Blimp 1 would be involved in Treg stabilization in the tumor, and targeting Blimp-1 in T cells would result in a decrease of Treg cells. Moreover, in a setting of inflammation, we previously demonstrated that IL-6 destabilizes T regs. In those settings, antagonizing IL-6 resulted in the induction of T regulatory cells producing IL-10 [45]. Although in this study we did not demonstrate directly that Blimp1 counteracts IL-6 in T regs, we demonstrated that the majority of T regs in PBMCs, which are Blimp1+, express IL-6R. Moreover, inducing Blimp1 in PBMCs was found to be associated with the induction of IL-6, but also IL-10 and TGFbeta, and a reduction of IL-2. All these elements indicate that Blimp1 upregulation was linked to immunosuppression in the presence of IL-6. In analyzing Blimp-1 levels in the lung of our human cohort with NSCLC, we observed that its expression was upregulated in the control and in the peri-tumoral areas in comparison to both control lungs and the tumoral areas. In the tumor area, there was also a reduction in CD4+ and CD8+ T-cell and Tbet mRNA levels. Furthermore, an analysis of Blimp1 expression in relation to tumor grade revealed an increase in expression at advanced cancer stages. At the protein level, an overall induction was observed in the tumoral area. Recently, the majority of studies have concentrated on the function of B cells in the tumor microenvironment, particularly in relation to tertiary lymphoid structures (TLS), which are located in close proximity to areas of follicular helper T cells (ThF) and B areas [35]. A significant accumulation of PD1-CD8+ exhausted T cells has been observed in the lungs of patients with NSCLC in tertiary lymphoid structures (TLS) in proximity to Th follicular cells and B cells [60]. Furthermore, Tfh cells are distinguished by the expression of PD1, which represents a key target for current immunotherapeutic approaches. The interaction between B and T cells is mediated by membrane receptors, including ICOS, CD40, PD1, and Slamf1. In addition to Blimp1, BCL6, and Slamf1 have also been identified as playing a role in Tfh cells. Consequently, we undertook an investigation into the expression of Slamf1 in the lungs of patients with NSCLC. This study revealed decreased Slamf1 expression in the tumor region, especially in the advanced stadium, where Blimp1 levels were elevated. Furthermore, correlation analysis with Blimp-1 demonstrated an inverse correlation, indicating that CD150 (Slamf1) can counter-regulate Blimp-1 levels. This hypothesis was corroborated by the observation that, following anti-CD150 treatment, Blimp1 expression was upregulated, accompanied by elevated levels of IL-6, IL-10, and TGFbeta 1. This is consistent with a positive prognostic role of Slamf1 in LUAD. Slamf1 likely marks a subtype of LUAD with better immune surveillance and outcomes [61]. Moreover, the *Human Protein Atlas*, using TCGA data, marks Slamf1 as a potential favorable prognostic marker in LUAD (*p* < 0.001) (en.wikipedia.org+15proteinatlas.org+15pmc.ncbi.nlm.nih.gov+15). Solid data in immunohistochemistry and transcriptomic analyses support high Slamf1 expression as linked to better survival. Moreover, although this study needs to be extended, it suggests that targeting Blimp1 by Slamf1 activation could improve current immunotherapy for lung cancer [53, 62].

## Supplementary information

Supplementary information

## Data Availability

No datasets were generated or analysed during the current study.

## References

[1] van Klaveren RJ. Lung cancer screening with low dose computed tomography: where do we stand today?. Eur J Cancer. 2009;45(Suppl 1):375–6. 19775636 10.1016/S0959-8049(09)70054-1

[2] Yang M, Mandal E, Liu FX, O’Hara RM Jr, Lesher B, Sanborn RE. Non-small cell lung cancer with MET amplification: review of epidemiology, associated disease characteristics, testing procedures, burden, and treatments. Front Oncol. 2023;13:1241402. 38273845 10.3389/fonc.2023.1241402 PMC10808753

[3] Neeve SC, Robinson BW, Fear VS. The role and therapeutic implications of T cells in cancer of the lung. Clin Transl Immunol. 2019;8:e1076. 10.1002/cti2.1076 PMC6712517 31485330

[4] Mountzios G, Naidoo J, Wang C, Creelan BC, Trotier DC, Campbell TC, et al. Beyond chemoimmunotherapy in advanced non-small cell lung cancer: new frontiers, new challenges. Am Soc Clin Oncol Educ Book. 2024;44:e432526. 38781566 10.1200/EDBK_432526

[5] Gridelli C, Peters S, Mok T, Garassino M, Ares LP, Attili I, de Marinis F, et al. Face to face among different chemo-immunotherapy combinations in the first line treatment of patients with advanced non-small cell lung cancer: results of an international expert panel meeting by the italian association of thoracic oncology (AIOT). Lung Cancer. 2024;187:107441. 38141488 10.1016/j.lungcan.2023.107441

[6] Borghaei H, Marinis F, Dumoulin D, Reynolds C, Theelen WSME, et al. SAPPHIRE Investigators SAPPHIRE: phase III study of sitravatinib plus nivolumab versus docetaxel in advanced nonsquamous non-small-cell lung cancer. Ann Oncol. 2024;35:66–76. 37866811 10.1016/j.annonc.2023.10.004

[7] He Y, Wu C, Svensson S, Almodovar-Abreu S, Rigaud B, McCollum E. Quantifying the effect of 4-dimensional computed tomography-based deformable dose accumulation on representing radiation damage for patients with locally advanced non-small cell lung cancer treated with standard-fractionated intensity-modulated radiation therapy. Int J Radiat Oncol Biol Phys. 2024;118:231–41. 37552151 10.1016/j.ijrobp.2023.07.016 PMC11379060

[8] Yu KR, Julliard WA. Sublobar resection of non-small-cell lung cancer: wedge resection vs. segmentectomy. Curr Oncol. 2024;31:2497–507. 38785468 10.3390/curroncol31050187 PMC11120128

[9] Bogatsa E, Lazaridis G, Stivanaki C, Timotheadou E. Neoadjuvant and adjuvant immunotherapy in resectable NSCLC. Cancers. 2024;16:1619. 10.3390/cancers16091619 PMC11083960 38730571

[10] Mazzaschi G, Facchinetti F, Missale G, Canetti D, Madeddu D, Zecca A. The circulating pool of functionally competent NK and CD8+ cells predicts the outcome of anti-PD1 treatment in advanced NSCLC. Lung Cancer. 2019;127:153–63. 30642544 10.1016/j.lungcan.2018.11.038

[11] Grant MJ, Herbst RS, Goldberg SB. Selecting the optimal immunotherapy regimen in driver-negative metastatic NSCLC. Nat Rev Clin Oncol. 2021;18:625–44. 34168333 10.1038/s41571-021-00520-1

[12] Li L, Chao QG, Ping LZ, Xue C, Xia ZY, Qian D, et al. The prevalence of FOXP3+ regulatory T-cells in peripheral blood of patients with NSCLC. Cancer Biother Radiopharm. 2009;24:357–67. 19538059 10.1089/cbr.2008.0612

[13] Ishibashi Y, Tanaka S, Tajima K, Yoshida T, Kuwano H. Expression of Foxp3 in non-small cell lung cancer patients is significantly higher in tumor tissues than in normal tissues, especially in tumors smaller than 30 mm. Oncol Rep. 2006;15:1315–9. 16596204

[14] Hendriks L, Besse B. New windows open for immunotherapy in lung cancer. Nature. 2018;558:376–7. 29907821 10.1038/d41586-018-05312-9

[15] Dubinett SM, Kradin RL. Cytokine immunotherapy of non-small cell lung cancer. Reg Immunol. 1993;5:232–43. 8240940

[16] Cascone T, Awad M, Spicer JD, He J, Lu S, Sepesi B, et al. Perioperative nivolumab in resectable lung cancer. N Engl J Med. 2024;390:1756–69. 38749033 10.1056/NEJMoa2311926

[17] Chaft JE, Dziadziuszko R, Haddock Lobo Goulart B. Moving immunotherapy into the treatment of resectable non-small cell lung cancer. Am Soc Clin Oncol Educ Book. 2024;44:e432500. 38788177 10.1200/EDBK_432500

[18] Reppert S, Boross I, Koslowski M, Türeci Ö, Koch S, Lehr HA, et al. A role for T-bet-mediated tumour immune surveillance in anti-IL-17A treatment of lung cancer. Nat Commun. 2011;2:600. 22186896 10.1038/ncomms1609

[19] Iwahori K, Uenami T, Yano Y, Ueda T, Tone M, Naito Y, et al. Peripheral T cell cytotoxicity predicts the efficacy of anti-PD-1 therapy for advanced non-small cell lung cancer patients. Sci Rep. 2022;12:17461. 36261600 10.1038/s41598-022-22356-0 PMC9582215

[20] Kallies A, Nutt SL. Terminal differentiation of lymphocytes depends on Blimp-1. Curr Opin Immunol. 2007;19:156–62. 17291741 10.1016/j.coi.2007.01.003

[21] Nutt SL, Fairfax KA, Kallies A. BLIMP1 guides the fate of effector B and T cells. Nat Rev Immunol. 2007;7:923–7. 17965637 10.1038/nri2204

[22] Kallies A, Xin A, Belz GT, Nutt SL. Blimp-1 transcription factor is required for the differentiation of effector CD8(+) T cells and memory responses. Immunity. 2009;31:283–95. 19664942 10.1016/j.immuni.2009.06.021

[23] Dixon ML, Leavenworth JD, Leavenworth JW. Lineage reprogramming of effector regulatory T cells in cancer. Front Immunol. 2021;12:717421. 34394124 10.3389/fimmu.2021.717421 PMC8355732

[24] Cretney E, Kallies A, Nutt SL. Differentiation and function of Foxp3(+) effector regulatory T cells. Trends Immunol. 2013;34:74–80. 23219401 10.1016/j.it.2012.11.002

[25] Ward-Hartstonge KA, McCall JL, McCulloch TR, Kamps AK, Girardin A, Cretney E, et al. Inclusion of BLIMP-1(+) effector regulatory T cells improves the Immunoscore in a cohort of New Zealand colorectal cancer patients: a pilot study. Cancer Immunol Immunother. 2017;66:515–22. 28116480 10.1007/s00262-016-1951-1 PMC11028880

[26] Dixon ML, Luo L, Ghosh S, Grimes JM, Leavenworth JD, Leavenworth JW, et al. Remodeling of the tumor microenvironment via disrupting Blimp1(+) effector Treg activity augments response to anti-PD-1 blockade. Mol Cancer. 2021;20:150. 34798898 10.1186/s12943-021-01450-3 PMC8605582

[27] Shen E, Rabe H, Luo L, Wang L, Wang Q, Yin J, et al. Control of germinal center localization and lineage stability of follicular regulatory T cells by the Blimp1 transcription factor. Cell Rep. 2020;31:107575. 32348761 10.1016/j.celrep.2020.107575 PMC7481879

[28] Mackay LK, Minnich M, Kragten NAM, Liao Y, Nota B, Seillet C, et al. Hobit and Blimp1 instruct a universal transcriptional program of tissue residency in lymphocytes. Science. 2016;352:459–63. 27102484 10.1126/science.aad2035

[29] Vahl JM, Friedrich J, Mittler S, Trump S, Heim L, Kachler K, et al. Interleukin-10-regulated tumour tolerance in non-small cell lung cancer. Br J Cancer. 2017;117:1644–55. 29016555 10.1038/bjc.2017.336 PMC5729436

[30] Heim L, Friedrich J, Engelhardt M, Trufa DI, Geppert CI, Rieker RJ, et al. NFATc1 promotes antitumoral effector functions and memory CD8(+) T-cell differentiation during non-small cell lung cancer development. Cancer Res. 2018;78:3619–33. 29691251 10.1158/0008-5472.CAN-17-3297

[31] Xiang Y, Liu X, Wang Y, Zheng D, Meng Q, Jiang L, et al. Mechanisms of resistance to targeted therapy and immunotherapy in non-small cell lung cancer: promising strategies to overcoming challenges. Front Immunol. 2024;15:1366260. 38655260 10.3389/fimmu.2024.1366260 PMC11035781

[32] Song L, Gong Y, Wang E, Huang J, Li Y. Unraveling the tumor immune microenvironment of lung adenocarcinoma using single-cell RNA sequencing. Ther Adv Med Oncol. 2024;16:17588359231210274. 38606165 10.1177/17588359231210274 PMC11008351

[33] Besse B, Pons-Tostivint E, Park K, Hartl S, Forde PM, Hochmair MJ, et al. Biomarker-directed targeted therapy plus durvalumab in advanced non-small-cell lung cancer: a phase 2 umbrella trial. Nat Med. 2024;30:716–29. 38351187 10.1038/s41591-024-02808-y PMC10957481

[34] Hiltbrunner S, Cords L, Kasser S, Freiberger SN, Kreutzer S, Toussaint NC, et al. Acquired resistance to anti-PD1 therapy in patients with NSCLC associates with immunosuppressive T cell phenotype. Nat Commun. 2023;14:5154. 37620318 10.1038/s41467-023-40745-5 PMC10449840

[35] Laumont CM, Banville AC, Gilardi M, Hollern DP, Nelson BH. Tumour-infiltrating B cells: immunological mechanisms, clinical impact and therapeutic opportunities. Nat Rev Cancer. 2022;22:414–30. 35393541 10.1038/s41568-022-00466-1 PMC9678336

[36] Xin A, Masson F, Liao Y, Preston S, Guan T, Gloury R, et al. A molecular threshold for effector CD8(+) T cell differentiation controlled by transcription factors Blimp-1 and T-bet. Nat Immunol. 2016;17:422–32. 26950239 10.1038/ni.3410 PMC5779087

[37] Sledzinska A, Vila de Mucha M, Bergerhoff K, Hotblack A, Demane DF, Ghorani E, et al. Regulatory T cells restrain interleukin-2- and Blimp-1-dependent acquisition of cytotoxic function by CD4(+) T cells. Immunity. 2020;52:151–166.e156. 31924474 10.1016/j.immuni.2019.12.007 PMC7369640

[38] Zundler S, Becker E, Spocinska M, Slawik M, Parga-Vidal L, Stark R, et al. Hobit- and Blimp-1-driven CD4(+) tissue-resident memory T cells control chronic intestinal inflammation. Nat Immunol. 2019;20:288–300. 30692620 10.1038/s41590-018-0298-5

[39] Fu SH, Yeh LT, Chu CC, Yen BL, Sytwu HK. New insights into Blimp-1 in T lymphocytes: a divergent regulator of cell destiny and effector function. J Biomed Sci. 2017;24:49. 28732506 10.1186/s12929-017-0354-8 PMC5520377

[40] Kallies A, Zehn D, Utzschneider DT. Precursor exhausted T cells: key to successful immunotherapy?. Nat Rev Immunol. 2020;20:128–36. 31591533 10.1038/s41577-019-0223-7

[41] Heim L, Yang Z, Tausche P, Hohenberger K, Ciriac MT, Koelle J, et al. IL-9 producing tumor-infiltrating lymphocytes and Treg subsets drive immune escape of tumor cells in non-small cell lung cancer. Front Immunol. 2022;13:859738. 35514957 10.3389/fimmu.2022.859738 PMC9065342

[42] Lu KT, Kanno Y, Cannons JL, Handon R, Bible P, Elkahloun AG, et al. Functional and epigenetic studies reveal multistep differentiation and plasticity of in vitro-generated and in vivo-derived follicular T helper cells. Immunity. 2011;35:622–32. 22018472 10.1016/j.immuni.2011.07.015 PMC3235706

[43] Memon D, Schoenfeld AJ, Ye D, Fromm G, Rizvi H, et al. Clinical and molecular features of acquired resistance to immunotherapy in non-small cell lung cancer. Cancer Cell. 2024;42:209–224 e209. 38215748 10.1016/j.ccell.2023.12.013 PMC11249385

[44] Yigit B, Halibozek PJ, Chen SS, O’Keeffe MS, Arnason J, Avigan D, et al. A combination of an anti-SLAMF6 antibody and ibrutinib efficiently abrogates expansion of chronic lymphocytic leukemia cells. Oncotarget. 2016;7:26346–60. 27029059 10.18632/oncotarget.8378 PMC5041984

[45] Doganci A, Eigenbrod T, Krug N, De Sanctis GT, Hausding M, Erpenbeck VJ, et al. The IL-6R alpha chain controls lung CD4+CD25+ Treg development and function during allergic airway inflammation in vivo. J Clin Invest. 2005;115:313–25. 15668741 10.1172/JCI22433 PMC544603

[46] Giunta, Barra EFG, De Falco V, Argenziano G, Napolitano S, Vitale P, et al. Baseline IFN-gamma and IL-10 expression in PBMCs could predict response to PD-1 checkpoint inhibitors in advanced melanoma patients. Sci Rep. 2020;10:17626. 33077770 10.1038/s41598-020-72711-2 PMC7573589

[47] Neumann C, Heinrich F, Neumann K, Junghans V, Mashreghi MF, Ahlers J, et al. Role of Blimp-1 in programing Th effector cells into IL-10 producers. J Exp Med. 2014;211:1807–19. 25073792 10.1084/jem.20131548 PMC4144744

[48] Doganci A, Sauer K, Karwot R, Finotto S. Pathological role of IL-6 in the experimental allergic bronchial asthma in mice. Clin Rev Allergy Immunol. 2005;28:257–70. 16129910 10.1385/CRIAI:28:3:257

[49] Garg G, Moreno H, Vasanthakumar A, Floess S, Lepennetier G, Oellinger R, et al. Blimp1 prevents methylation of Foxp3 and loss of regulatory T cell identity at sites of inflammation. Cell Rep. 2019;26:1854–1868.e1855. 30759395 10.1016/j.celrep.2019.01.070 PMC6389594

[50] Lu P, Youngblood BA, Austin JW, Mohammed AU, Butler R, Ahmed R, et al. Blimp-1 represses CD8 T cell expression of PD-1 using a feed-forward transcriptional circuit during acute viral infection. J Exp Med. 2014;211:515–27. 24590765 10.1084/jem.20130208 PMC3949569

[51] Li, Zhang QL, You W, Xu J, Dai J, Hua D, et al. PRDM1/BLIMP1 induces cancer immune evasion by modulating the USP22-SPI1-PD-L1 axis in hepatocellular carcinoma cells. Nat Commun. 2022;13:7677. 36509766 10.1038/s41467-022-35469-x PMC9744896

[52] Doganci A, Karwot R, Maxeiner JH, Scholtes P, Schmitt E, Neurath MF, et al. IL-2 receptor beta-chain signaling controls immunosuppressive CD4+ T cells in the draining lymph nodes and lung during allergic airway inflammation in vivo. J Immunol. 2008;181:1917–26. 18641329 10.4049/jimmunol.181.3.1917

[53] Chow MT, Ozga AJ, Servis RL, Frederick DT, Lo JA, Fisher DE, et al. Intratumoral activity of the CXCR3 chemokine system is required for the efficacy of anti-PD-1 therapy. Immunity. 2019;50:1498–1512 e1495. 31097342 10.1016/j.immuni.2019.04.010 PMC6527362

[54] Mami-Chouaib F, Blanc C, Corgnac S, Hans S, Malenica I, Granier C, et al. Resident memory T cells, critical components in tumor immunology. J Immunother Cancer. 2018;6:87. 30180905 10.1186/s40425-018-0399-6 PMC6122734

[55] Rubtsov YP, Niec RE, Josefowicz S, Li L, Darce J, Mathis D, et al. Stability of the regulatory T cell lineage in vivo. Science. 2010;329:1667–71. 20929851 10.1126/science.1191996 PMC4262151

[56] DuPage M, Chopra G, Quiros J, Rosenthal WL, Morar MM, Holohan D, et al. The chromatin-modifying enzyme Ezh2 is critical for the maintenance of regulatory T cell identity after activation. Immunity. 2015;42:227–38. 25680271 10.1016/j.immuni.2015.01.007 PMC4347854

[57] Williams LM, Rudensky AY. Maintenance of the Foxp3-dependent developmental program in mature regulatory T cells requires continued expression of Foxp3. Nat Immunol. 2007;8:277–84. 17220892 10.1038/ni1437

[58] Cretney, Xin EA, Shi W, Minnich M, Masson F, Miasari M, et al. The transcription factors Blimp-1 and IRF4 jointly control the differentiation and function of effector regulatory T cells. Nat Immunol. 2011;12:304–11. 21378976 10.1038/ni.2006

[59] Tang RJ, Shen SN, Zhao XY, Nie YZ, Xu YJ, Ren J, et al. Mesenchymal stem cells-regulated Treg cells suppress colitis-associated colorectal cancer. Stem Cell Res Ther. 2015;6:71. 25889203 10.1186/s13287-015-0055-8 PMC4414289

[60] Xiong D, Wu YB, Jin C, Li JJ, Gu J, Liao YF, et al. Elevated FUS/TLS expression is negatively associated with E-cadherin expression and prognosis of patients with non-small cell lung cancer. Oncol Lett. 2018;16:1791–1800. 30008867 10.3892/ol.2018.8816 PMC6036447

[61] Zhang Z, Zhang P, Xie J, Cui Y, Wang S, Yue D, et al. Five-gene prognostic model based on autophagy-dependent cell death for predicting prognosis in lung adenocarcinoma. Sci Rep. 2024;14:26449. 39488588 10.1038/s41598-024-76186-3 PMC11531468

[62] Kang MJ, Kim KM, Bae JS, Park HS, Lee H, Chung MJ, et al. Tumor-infiltrating PD1-positive lymphocytes and FoxP3-positive regulatory T cells predict distant metastatic relapse and survival of clear cell renal cell carcinoma. Transl Oncol. 2013;6:282–9. 23730407 10.1593/tlo.13256 PMC3660796

