# Supplementary material for: The transcription factor Blimp-1 is suppressed by SLAMF1 and drives Treg cell-mediated immune evasion in non-small cell lung cancer
Source: BJC Rep. 2025 Oct 21;3:74. doi: 10.1038/s44276-025-00184-9 (PMC12540759; doi:10.1038/s44276-025-00184-9)
Supplement: Supplementary file 1 — Supplementary information [file 44276_2025_184_MOESM1_ESM.pdf]

The transcription factor Blimp-1 is suppressed by SLAMF1 and drives Treg cell-mediated immune evasion in non-small cell lung cancer.

Susetta Finotto[1,2,3,4*], Denis I. Trufa[3,4,5], Sonja Trump[1], Laura Neurath[1], Katja Hohenberger [1], Patrick Tausche [1], Nicole Neurath[1], Sviatoslav Tsiumpala[1], Susanne Mittler[1], Andreas Wild [6], Elvedina Nendel[1], Horia Sirbu[3,4,5] and Arndt Hartmann[3,4,7].

[1] Department of Molecular Pneumology, Friedrich Alexander University Erlangen-Nürnberg (FAU), Universitätsklinikum Erlangen, 91054 Erlangen, Germany.

[2] Deutsches Zentrum für Immuntherapie (DZI), Erlangen, Germany,

[3] Bavarian Cancer Research Center (BZKF), Erlangen, Germany,

[4] Comprehensive Cancer Center Erlangen-EMN (CCC ER-EMN), Erlangen, Germany

[5] Department of Thoracic Surgery, University Hospital, Friedrich-Alexander-Universität Erlangen-Nürnberg, Erlangen, Germany

[6] Department of Immune Modulation, Dermatology Clinic, Friedrich Alexander University Erlangen-Nürnberg (FAU), Universitätsklinikum Erlangen, 91054 Erlangen, Germany

[7] Institute of Pathology, University Hospital, Friedrich-Alexander-Universität Erlangen-Nürnberg, Erlangen, Germany

***Correspondence:**

Prof. Dr. Dr. Susetta Finotto

Universitätsklinikum Erlangen

Abt. Molekulare Pneumologie

Hartmannstraße 14

91052 Erlangen

Phone: +49-9131-85-35883

“The authors declare no competing interests.”

-

Abbreviations used

ADC= adenocarcinoma

SCC= squamous carcinoma

HC= healthy controls

CN= Control lung from subjects that underwent lung surgery for reasons different from lung cancer.

CTR= control region of the lung from subjects that underwent lung surgery after a diagnosis of lung cancer.

PT= peritumoral tissue

TU= tumor

G1,G2, G3= tumor grade

MTS= Metastasis

Table S1 Characteristics of the patients analyzed in this study

Patient Code	Histological classification	Grading	Tumor Diameter (cm)	TNM-Stadium
3-MP	ADC	G3	5	IIA
6-MP	MTS ADC Gut	G3	4	IV(Rectum-ADC)
9-MP	ADC	G2	2,7	IIIA
15-MP	ADC	G3	2,5	IA3
16-MP	ADC	G3	1,8 2,8	IIB
17-MP	ADC	G2	2,6	IA3
18-MP	MTS ADC	G2	6,4	IV (Sigma-Carcinoma)
20-MP	ADC	G3	2,7	IA3
23-MP	ADC	G2	4,5	IIA
34-MP	ADC	G2	1,8	IA2
44-MP	ADC	G1	1,5	IA2
53-MP	ADC	G2	1,4	IA2
56-MP	ADC	G2	4	IB
77-MP	ADC	G2	0,9	IA1
81-MP	ADC	G3	1,6	IA2
94-MP	ADC	G2	2,2	IA3
98-MP	ADC	G3	2,9	IIIA
99-MP	ADC	G3	3,5 und 1,5	IIIA
107-MP	ADC	G1	1,7	IA2
118-MP	ADC	G1	1,5	IA2
91-TU	ADC	G2	3,6	IIIA
107-TU	ADC	G1	1,7	IA2
97-TU	ADC	G2	5	IIB
108-TU	ADC	G2	3,6	IB
110-TU	ADC	G3	7,2	IIIB
112-TU	ADC	G2	1,6	IIIB
102-TU	ADC	G2	2,1	IIB
103-TU	ADC	G3	3,9	IB
68-TU	ADC	G3	8,5	IIA
105-TU	ADC	G3	1,8	IA2
69-TU	ADC	G2	3,1	IB
106-TU	ADC	G2	1,2	IA2
83-TU	ADC	G3	5,5	IVA
55-TU	ADC	G2	1,8	IIIA
62-TU	ADC	G2	3,5	IB
80-TU	ADC	G3	5,4	IIB
26-TU	ADC	G3	1,3	IVA
51-TU	ADC	G3	2,3	IVA
146-TU	ADC	G1	1,8	IA
159-TU	ADC	?	?	III
169-TU	ADC	G2	3,3	IB
149-TU	ADC	G2	1,2	IA2
160-TU	ADC	G2	1,7	IA2
173-TU	ADC	G2	2,9	IB
151-TU	ADC	G1	4,2	IIA
164-TU	ADC	G3	3,1	IB
175-TU	ADC	G2	2,1	IB

155-TU	ADC	G2	1,3	IA2
165-TU	ADC	G2	1,7	IA2
177-TU	ADC	G3	2,9	IA3

ADC=Adenocarcinoma; MTS= Metastasis; MTS ADC = adenocarcinoma with metastasis. ADC-Rektum, Sigma= Gut adenocarcinoma; The **classification of lung cancer in grading** refers to how abnormal the cancer cells look under a microscope and how quickly the tumor is likely to grow and spread. This is different from **staging**. The terms **Tumor IA, IIA**, etc, are **cancer staging classifications**, specifically referring to **Stage I** and **Stage II** tumors, often used in systems like the **TNM staging system** by the **American Joint Committee on Cancer (AJCC)** or other organ-specific staging criteria. It describes the extent of cancer in the body.

T-primary tumour: 0: 1a: Tumour 2 cm or less in greatest dimension; 1b: Tumour more than 2 cm but not more than 3 cm in greatest dimension; 2a: Tumour more than 3 cm but not more than 5 cm in greatest dimension; 2b: Tumour more than 5 cm but not more than 7 cm in greatest dimension; 3: Tumour more than 7 cm;

Grading helps guide treatment and gives insight into prognosis. Non-Small Cell Lung Cancer (NSCLC) – Grading System

NSCLC includes adenocarcinoma, squamous cell carcinoma, and large cell carcinoma.

- Grade 1 (Low grade / Well-differentiated)
  - Cells look more like normal lung cells
  - Grow and spread more slowly
  - Generally better prognosis
- Grade 2 (Intermediate grade / Moderately differentiated)
  - Cells look somewhat abnormal
  - Moderate growth and spread
- Grade 3 (High grade / Poorly differentiated)
  - Cells look very abnormal and different from normal cells
  - Tend to grow and spread more quickly
  - Worse prognosis
- Grade 4 (Undifferentiated) (less commonly used)
  - Cells do not resemble normal cells at all
  - Very aggressive behavior

Table S2. Characteristics of the patients analyzed in this study by western blot analysis

Patient Code	Histological classification	Grading	Tumor Diameter (cm)	TNM-Stadium
9-MP	ADC	G2	2,7	IIIA
15-MP	ADC	G3	2,5	IA3
17-MP	ADC	G2	2,6	IA3
6-MT				
18-MP	MTS ADC	G2	6,4	IV (Sigma-Ca)
11-MP	Inflammation			
34-MP	ADC	G2	1,8	IA2
26-MP				
44-MP	ADC	G1	1,5	IA2
77-MP	ADC	G2	0,9	IA1
81-MP	ADC	G3	1,6	IA2
94-MP	ADC	G2	2,2	IA3
107-MP	ADC	G1	1,7	IA2
118-MP	ADC	G1	1,5	IA2

Western Blot analysis

Western blot in **Figure 1d** (upper blot) (27.05.2020)

**Lane**	**Blimp-1**	**total protein**	**Blimp1/Protein**		**Patient**
**Label**	**Adj. Vol. (Int)**	**Adj. Total Lane Vol. (Int)**			
U1	14.893.670,94	95134104	0,15655449	CN	MP-6
U2	55.971.925,16	80703810	0,69354749	CN	MP-9
U3	35.584.774,00	61542615	0,57821355	CTRG1	MP-107
U4	32.338.824,80	72873255	0,44376808	CTRG1	MP-118
U5	38.908.607,10	129905370	0,299515	CTRG2	MP-77
U6	15.767.287,51	100169420	0,1574062	CTRG2	MP-94
U7	4.821.463,98	75063555	0,06423176	CTRG3	MP-15

U8	14.019.222,94	89264837	0,15705202	CTRG3	MP-81
U9	63.149.479,92	55873686	1,13021861	TUG1	MP-107
U10	30.935.026,29	54294768	0,56976072	TUG1	MP118
U11	101.709.302,84	55438096	1,83464639	TUG2	MP-77
U12	121.748.802,82	123510372	0,98573748	TUG2	MP-94
U13	7.712.976,49	40984664	0,18819177	TUG3	MP-15
U14	26.562.427,69	65561458	0,4051531	TUG3	MP-81

Western blot analysis **Figure 1d** middle blot

Lane No.	Blimp	Adj. Total Lane Vol. (Int)	Blimp/total protein	Patient	
1	1.876.174,57	723020460	0,002594912	CTR G2	MP53
2	919.260,04	494363776	0,001859481	CTR G2	MP56
3	513.378,35	570565452	0,000899771	CTR G3	MP3
4	1.328.442,22	353094596	0,003762284	CTR G3	MP20
5	420.118,48	489150327	0,000858874	PT G3	MP3
6	687.121,91	260202508	0,00264072	PT MTS	MP18
7	19.318.415,48	285342455	0,067702563	A549	
Standard					
8	888.044,52	295134448	0,003008949	PT G2	MP53
9	483.487,13	321337884	0,001504607	PT G2	MP56
10	722.319,78	297868375	0,002424963	PT G3	MP20
11	1.873.342,26	460435878	0,004068628	TU G2	MP53
12	4.395.046,83	422261125	0,010408362	TU G2	MP56
13	1.339.645,65	424037250	0,003159264	TU G3	MP3
14	3.322.917,57	541047092	0,006141642	TU G3	MP20

GAPDH Western blot analysis **Figure 1d** middle blot (same membrane as above)

	**Blimp-1**	**GAPDH**		**Patient**
Lane No.	Blimp band	GAPDH band	Blimp/GAPDH	
1	1.876.174,57	27.567.265,54	0,068058058	CTR G2 53
2	919.260,04	73.637.448,47	0,012483594	CTR G2 56
3	513.378,35	43.606.140,86	0,011773075	CTR G3 3
4	1.328.442,22	64.325.913,34	0,020651743	CTR G3 20
5	420.118,48	91.529.169,98	0,004589995	PT G3 3
6	687.121,91	61.577.991,89	0,011158563	PT MTS 18
7	19.318.415,48	131.118.286,39	0,147335784	A549
Standard				
8	888.044,52	75.693.196,87	0,011732158	PT 53
9	483.487,13	38.320.026,25	0,012617088	PT 56
10	722.319,78	45.861.283,89	0,015750099	PT20
11	1.873.342,26	70.115.322,42	0,026718015	TU 53
12	4.395.046,83	82.526.626,17	0,053256107	TU 56
13	1.339.645,65	78.453.307,16	0,017075707	TU 3
14	3.322.917,57	122.062.522,09	0,027223078	TU20

Western blot lower blot Figure 1d

**Lane**	**Blimp-1**	**total protein**	**Blimp1/protein**	**Patient**
**Label**	**Adj. Vol. (Int)**	**Adj. Total Lane Vol. (Int)**		
U1	1.576.783,03	370545552	0,0042553	CN1
U2	2.074.269,08	397719120	0,00521541	CN3
U3	643.915,19	560048500	0,00114975	CTR G1 44
U4	324.199,47	508675926	0,00063734	CTR G2 9
U5	342.171,63	439725270	0,00077815	CTR G2 17
U6	210.782,49	403955200	0,0005218	CTR G3 15
U7	270.276,10	231958573	0,00116519	CTR G3 MT 26
U8	229.053,19	160359936	0,00142837	PT G2 9
U9	739.880,53	300404286	0,00246295	PT G2 17
U10	802.981,97	345143436	0,00232652	PT G3 15
U11	481.084,05	414290765	0,00116122	PT G3 MT 26
U12	2.716.275,25	421587712	0,00644297	TU G2 9
U13	751.149,42	454241436	0,00165363	TU G3 15

U14	166.833,95	243131846	0,00068619	TU G3 MT TU26

U= sample number

Table S3: Characteristics of the patients analyzed in this study by Multiplex analysis of PBMCs supernatants

Patient Code	Histological classification	Grading	Tumor Diameter (cm)	TNM-Stadium
136	ADC			?
141	ADC	G2	3,8	IB
143	ADC	G2	2,7 (x2)	IIIA
150-MP	ADC	G2	2,3	IA3
152-MP	ADC-neuroendocrines Ca	G3	3,2	IB
153-MP	SCC	G3	12	IIIA
154-MP				
155-MP	ADC	G3	1,3	IA2
157-MP	ADC	G3	2,5	IIIA
158-MP	SCC	G2	1,4	IV
159-MP	ADC			III
169	ADC	G2	2,1	IA3
177	ADC	G3	2,9	IA3
178	ADC	G3	1,2	IA2
181	SCC	G3	8,5	IIB
170	SCC	G3	2,1	IA3
174	ADC-SCC	G3	1,5	IA2
175	ADC	G2	2,1	IB
176	SCC	G3	2,7	IA3

T-primary tumour: 0: 1a: Tumour 2 cm or less in greatest dimension; 1b: Tumour more than 2 cm but not more than 3 cm in greatest dimension; 2a: Tumour more than 3 cm but not more than 5 cm in greatest dimension; 2b: Tumour more than 5 cm but not more than 7 cm in greatest dimension; 3: Tumour more than 7 cm;

Abbreviations used

Table S4. Clinical data of the cohort of control subjects whose lung was analysed in this study.

Sample ID	Histological Classification	Tumour Ø (cm)	Grading	T N M	TNM Stadium	Average Smoking (P/Y)
K1	Pneumothorax	0	/	/ / /	/	#
K2	Chondroid Hamartoma	0	/	/ / /	/	#
K3	Pneumothorax	0	/	/ / /	/	#
K4	Chondromatous Hamartoma	0	/	/ / /	/	#
K5	Pneumothorax	0	/	/ / /	/	#
K6	Rheumatoid Granuloma	0	/	/ / /	/	#
K7	Sarcoidosis	0	/	/ / /	/	#
K8	Pneumothorax	0	/	/ / /	/	#
K9	Chondroid Hamartoma	0	/	/ / /	/	#
K10	Pneumothorax	0	/	/ / /	/	#

Abbreviations: # No information available; / non-existing.

Table S5: List of subject analyzed in PBMCs studies by FACS analysis.

<table>
<tr><th>Nr.</th><th>Patient</th><th>ADC/SCC</th><th>Grading</th></tr>
<tr><td>HC 1</td><td>iK3</td><td rowspan="5" colspan="2">Healthy Controls</td></tr>
<tr><td>HC 2</td><td>iK1</td></tr>
<tr><td>HC 3</td><td>iK4</td></tr>
<tr><td>HC 4</td><td>iK9</td></tr>
<tr><td>HC 5</td><td>C-508</td></tr>
<tr><td>NSCLC 1</td><td>MP-149</td><td>ADC</td><td>G2</td></tr>
<tr><td>NSCLC 2</td><td>MP-150</td><td>ADC</td><td>G2</td></tr>
<tr><td>NSCLC 3</td><td>MP-152</td><td>ADC</td><td>G3</td></tr>
</table>

NSCLC 4	MP-153	SCC	G3
NSCLC 5	MP-155	ADC	G2

HC=Healthy controls; NSCLC=non small cell lung cancer.

Table S6: List of control subject analyzed in IL-6R study

AZCRA-Study Subject code	Weight (kg)	Height cm
**525**	**62**	**171**
**530**	**86**	**172**
**565**	**88**	**184**

Table S7: List of control subject analyzed in anti CD150 and anti PD1 antibodies

Subject	Weight (Kg)	Height (cm)
1	64	163
2	55	170
3	57	157
4	59	175

Table S8: List of antibodies used for Flow Cytometry.

Antigene	Conjugate	Reactivity	Dilution	Company	Cat#
Blimp-1	Clone 6D3	Human	1:50	BD Biosciences	564702
CD3	APC-Fire750	Human	1:200	BioLegend	300470
CD3e	FITC	Human	1:200	R&D Systems	FAB100F
CD4	PerCP	Human	1:200	BioLegend	317432
CD8	APC-Cy7	Human	1:200	BioLegend	344714
CD8	PE	Human	1:200	BioLegend	300908
CD25	PE-Cy7	Human	1:100	BioLegend	302612
FoxP3	AlexaFluor 647	Human	1:50	BioLegend	320214
T-bet	BV421	Human	1:50	BioLegend	644832

IL-6R	UV4	Human	1:100	BioLegend	352809

Supplementary Table S9: List of primers used for Quantitative Real-Time PCR.

Species	Gene	Primer Sequence Eurofins Genomic
	T-bet	For 5‘-CCG TGA CTG CCT ACC AGA-3‘<br>Rev 5’-AAC AGG ATA CTG GGT GGG TAG GA-3‘
	CD4	For 5- AAC CTG GTG GTG ATG AGA GC<br>Rev- CTC AGC AGA CAC TGC CAC AT
	CD8	For 5- TCA CCC TTT ACT GCA ACC AC-3‘<br>Rev-5‘- TTG TCT CCC GAT TTG ACC AC-3‘
Human	*Slamf1*	For: 5´-ATG TTG CTG CCA CAG AGC CTG T-3‘<br>Rev: 5‘-CCT TTG TTG GTC TCT GGT GTC AG-3‘
	Perforin	For 5- GGA CCA GTA CAG CTT CAG CAC TG<br>Rev 5‘ AGT CAG GGT GCA GCG GG
	HPRT	For: 5′-TGA CAC TGG CAA AAC AAT GCA-3\`<br>Rev: 5′-GGT CCT TTT CAC CAG CAA GCT-3\`
	GAPDH	For: 5′-AAA TCA AGT GGG GCG ATG CT-3‘<br>Rev:5‘-CAA ATG AGC CCC AGC CTT CT-3‘
	Prdm1	BioRad Cat n . 10025637 sequence unknown

Supplementary figure

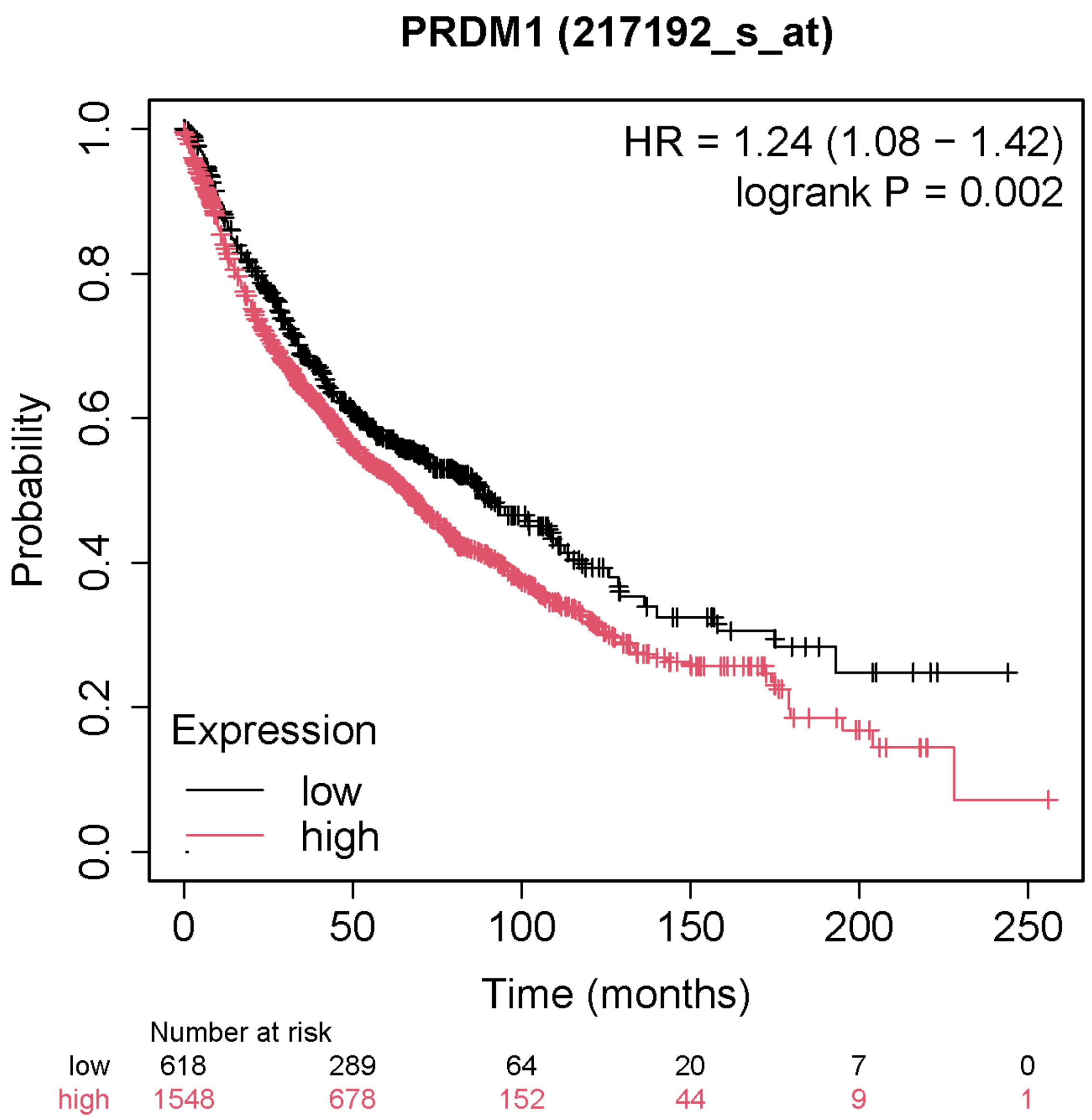

Figure S1. Kaplan Meier curve for Blimp1 gene. The data were recovered by using the Kaplan Meier Plotter online.2166 patients were analyzed. The difference between the 2 groups was statistically significant (p=0.002) between lower expression and high expression of Blimp1. The predicted survival was 88,7 months for the low expressing versus 65,57 months for high expressing Blimp1.
